# Editorial: Genetic disorders and rare diseases: *in vitro* models for preclinical pharmacological studies and translation

**DOI:** 10.3389/fphar.2023.1346648

**Published:** 2023-12-21

**Authors:** Shabir Hassan, Museer A. Lone

**Affiliations:** ^1^ Department of Biology, Khalifa University, Abu Dhabi, United Arab Emirates; ^2^ Advanced Materials Chemistry Center, Khalifa University, Abu Dhabi, United Arab Emirates; ^3^ Institute of Clinical Chemistry, University Hospital Zurich, University of Zurich, Zurich, Switzerland

**Keywords:** rare disease (RD), genetic disease, high throughput screen (HTS), preclinical (*in vivo*) studies, organoids, rabies virus (RABV), chemotherapy

The study and treatment of genetic disorders and rare diseases are profoundly complex, primarily due to their diverse genetic origins and low prevalence. This complexity necessitates innovative approaches in preclinical pharmacological studies, where *in vitro* models stand at the forefront of research and therapeutic development. These models serve as pivotal tools in understanding disease mechanisms and in the screening and development of potential treatments.

High Throughput Screening (HTS) allows relatively fast chemical, genetic, or pharmacological tests through automation, miniaturized assays, and large-scale data analysis to quickly identify active compounds, antibodies, or genes that modulate molecular pathways in question. HTS accelerates drug discovery by identifying candidates that affect a particular biological target or a disease process.

The emergence of induced pluripotent stem cells (iPSCs) and three dimensional (3D) culture technologies has marked a significant advancement in this field. Patient derived iPSCs enable the creation of patient-specific cell lines. These lines accurately mirror the disease phenotype at the cellular level, allowing for a relatively precise evaluation of drug responses and toxicity in the genetic context of the patient. This approach not only enhances the relevance of preclinical findings but also paves the way for more personalized therapeutic strategies. However, two-dimensional (2D) culture systems, where cells are grown on a flat surface often fail to mimic the natural tissue microenvironment, that may affect cell signaling pathways ultimately impeding drug discovery. In 3D culture systems, coherent cells within an extracellular matrix reflect better the physiological behavior as in an actual tissue environment.

In the article Research Topic on, “*Genetic Disorders and Rare Diseases: In Vitro Models for Preclinical Pharmacological Studies and Translation*”, the studies presented have utilized the combination of some of these methods to facilitate a deeper understanding of disease pathogenesis and aid in the identification of novel therapeutic targets, providing a scalable and controllable environment for rigorous pharmacological testing. This aspect is crucial for rare diseases, where patient availability for clinical trials is often limited. This approach underscores a paradigm shift in how these diseases are studied and treated, highlighting the importance of tailored research methodologies in addressing the unique challenges they present.

Drug repurposing involves finding new uses for existing drugs. For economic feasibility, repurposing can be especially important in addressing rare diseases or pathologies of low incidences. Combining drug repurposing with HTS allows efficient path to clinical application of existing drugs. Despite effective vaccines being available for pre- and post-exposure prophylaxis, there is no known effective treatment for rabies once symptoms have developed, at which point it is almost always fatal (Brunker and Mollentze, 2018). Caused by Rabies Virus (RABV), it remains a significant public health Research Topic, especially in resource-limited countries. Wu et al. utilized a pseudovirus system to establish a HTS screening assay for anti-RABV drug identification from FDA-approved compounds. An important addition to HTS is the assessment of investigative cellular processes using reporter systems. The authors used a pseudogene containing luciferase reporter enveloped by RABV glycoprotein (pRABV). The authors performed staggered testing, finally identifying 11 compounds with low cytotoxicity but high anti-pRABV efficacy. Final testing with a virulent strain, RABV CVS the group identified Clofazimine (CFZ), a riminophenazine used for the treatment of leprosy and tuberculosis, effective against RABV infection. CFZ has low solubility. Wu et al. tested the efficacy of CFZ and a series of soluble salt derivatives in mice post RABV infection. Both CFZ and some CFZ-derivatives delayed symptom onset and improved survival in rodents after intravascular administration of CVS strain. Although clinical testing is required to determine efficacy of CFZ or CFZ derivatives in humans, the study by Wu et al., demonstrates amply that drug repurposing can be achieved with intelligent experimental set-ups.

Anticancer drugs present relatively high rates of failures at clinical trials (Hutchinson and Kirk, 2011). In addition to the limitations of cell-culture models, genetic differences between preclinical animal models and human patients play major role in underscoring the actual efficacy of these molecules. Small-cell lung cancer (SCLC) accounts for about 10% of lung cancer cases in the US, and is known for its poor prognosis and high relapse rate after therapy. Sen et al. developed and used a functionalized alginate microbead model to replicate the lung alveolar structure. They co-cultured SCLC cell lines with primary adult lung fibroblasts (ALF) within this scaffold. Remarkably, the SCLC cells in this model not only proliferated rapidly but also invaded the microbead scaffold developing into tumors. Biophysical studies showed that these tumors displayed increased stiffness that increased over time and expressed SCLC specific markers. An important outcome of the study is that cell interactions are essential for cancer cell survival after drug treatment. SCLC cells grown alone show higher toxicity and decreased recuperation after treatment with anti-cancer drugs, Cisplatin and Etoposide than those that are co-cultured with ALF’s. This indicates model’s capability to mimic the resilience and regrowth of SCLC post-chemotherapy.

Drug-induced nephrotoxicity refers to kidney damage caused by medication or chemical exposure. Antibiotics (aminoglycosides), chemotherapy agents, heavy-metal exposure, and non-steroidal anti-inflammatory drugs (NSAIDs) induce nephrotoxicity and renal failure (Radi, 2019). Age, as well as metabolic diseases such as diabetes and hypertension predispose patients to nephrotoxicity (Khan et al., 2017). Identifying potential nephrotoxic effects necessitates rigorous preclinical testing and clinical trials to evaluate renal toxicity during drug development. Susa et al. report iPSC-derived kidney organoid models for assessing drug toxicity *in vitro*. These organoids express key proximal tubule drug transporters like anion/cation transporters OAT1, OAT3, and OCT2 at physiological levels and patterns comparable to those found in kidney. The study also highlights the potential of kidney organoids in assessing drug-induced damage. Additionally, the use of a genetically encoded biosensor, Perceval HR for ATP and ADP, suggests an efficient method for high-throughput drug toxicity screening. The model developed by Susa et al. facilitates real-time observation of nephrotoxicity mimicking drug-induced tubular and glomerular injury in a segment-specific manner, enhancing the effectiveness and efficiency of high-throughput drug screening.
